# Molecular adaptations to phosphorus deprivation and comparison with nitrogen deprivation responses in the diatom *Phaeodactylum tricornutum*

**DOI:** 10.1371/journal.pone.0193335

**Published:** 2018-02-23

**Authors:** Leila Alipanah, Per Winge, Jens Rohloff, Javad Najafi, Tore Brembu, Atle M. Bones

**Affiliations:** Cell, Molecular Biology and Genomics Group, Department of Biology, Norwegian University of Science and Technology, Trondheim, Norway; University of Connecticut, UNITED STATES

## Abstract

Phosphorus, an essential element for all living organisms, is a limiting nutrient in many regions of the ocean due to its fast recycling. Changes in phosphate (P_i_) availability in aquatic systems affect diatom growth and productivity. We investigated the early adaptive mechanisms in the marine diatom *Phaeodactylum tricornutum* to P deprivation using a combination of transcriptomics, metabolomics, physiological and biochemical experiments. Our analysis revealed strong induction of gene expression for proteins involved in phosphate acquisition and scavenging, and down-regulation of processes such as photosynthesis, nitrogen assimilation and nucleic acid and ribosome biosynthesis. P deprivation resulted in alterations of carbon allocation through the induction of the pentose phosphate pathway and cytosolic gluconeogenesis, along with repression of the Calvin cycle. Reorganization of cellular lipids was indicated by coordinated induced expression of phospholipases, sulfolipid biosynthesis enzymes and a putative betaine lipid biosynthesis enzyme. A comparative analysis of nitrogen- and phosphorus-deprived *P*. *tricornutum* revealed both common and distinct regulation patterns in response to phosphate and nitrate stress. Regulation of central carbon metabolism and amino acid metabolism was similar, whereas unique responses were found in nitrogen assimilation and phosphorus scavenging in nitrogen-deprived and phosphorus-deprived cells, respectively.

## Introduction

Diatoms are unicellular photosynthetic microalgae responsible for approximately 40% of marine primary productivity in the ocean [1]. Diatoms are major contributors to the “biological carbon pump” via the sedimentation of dead cells to the sea floor resulting in the sequestration of CO_2_ from the atmosphere to the deep oceans, thereby playing a crucial role in the global carbon cycle [2]. Diatom genomes contain genes and pathways acquired from different sources through secondary endosymbiosis and horizontal gene transfer from bacteria [2]. This provides diatoms with metabolic properties that help them survive adverse conditions such as nutrient deprivation.

Diatom growth can be limited by nutrients such as nitrogen (N), silicon (Si), phosphorus (P) and iron (Fe) [3]. Phosphorus is an essential component of nucleic acids, phospholipids and intermediate metabolites, and its availability can affect primary production and the carbon cycle in aquatic environments [4]. P recycling is very fast in the ocean, and P limitation has been observed in many regions of the open ocean [5]. The most common soluble inorganic form of P taken up by phytoplankton is orthophosphate anions (P_i_). Diatoms are able to increase their P uptake in response to P deficiency [6]. Induction of phosphate transporters and alkaline phosphatases, which are associated with an increase in the capacity of P uptake in diatoms, have been reported in P limitation studies [7, 8]. Diatoms are also able to use dissolved organic P (DOP) under P-limited conditions. Unlike P_i_, DOP cannot be assimilated directly by diatoms; instead, they possess several scavenging enzymes besides alkaline phosphatase for the utilization of DOP, such as phosphodiesterases and nucleotidases [9, 10]. Changes in the function of the photosynthetic apparatus is another effect of P deprivation observed in diatoms and other phytoplankton [11, 12].

To cope with P limitation, diatoms modulate several metabolic pathways. Dyhrman et al. [7] reported modifications in the expression of enzymes related to the glycolytic pathway and translation in *Thalassiosira pseudonana*. Similar responses have been observed in the brown tide alga *Aureococcus anophagefferens* under P stress [13]. Phosphorus limitation in *Phaeodactylum tricornutum* resulted in a higher level of lipids, primarily triacylglycerols (TAG) [14]. As a function of P limitation, phytoplankton replace phospholipids with non-phosphorus membrane lipids to reduce their P demand [15]. *T*. *pseudonana* grown under P-limited conditions was reported to substitute phospholipids with sulfolipids [16].

*P*. *tricornutum* is a model diatom for large-scale molecular analyses due to its published genome sequence and a well-developed molecular toolkit [17–20]. *P*. *tricornutum* was used to elucidate physiological and biochemical adaptations to P deficiency in diatoms. Transcriptional profiles of P-deprived and P replete cultures at 48 h and 72 h were combined with metabolite profiling and physiological measurements. We identified essential metabolic pathway remodelling under P deficiency. A combination of metabolite and gene expression data provided details explaining the observed changes in the lipid composition of P deprived cultures. In addition, a comparative analysis of nitrogen- and phosphorus-deprived *P*. *tricornutum* showed similar, opposite and unique regulation patterns in response to phosphate and nitrate stress.

## Materials and methods

### Growth conditions

Axenic cultures of *P*. *tricornutum* clone Pt1 8.6 (CCMP632) were incubated in f/2 medium containing 0.2 μm filtered autoclaved seawater enriched with filter-sterilized macro and micronutrients according to Guillard [21]. The cultures were kept in exponential growth at 15°C with stirring at 120 rpm for mixing and oxygenation under continuous white fluorescent light of approximately 60 μmol photons m^-2^ s^-1^ in an incubator shaker (Innova 4340, New Brunswick Scientific, Enfield, CT, USA) for three weeks to be acclimated. The cultivation temperature was chosen to avoid control cultures entering stationary growth phase within the experimental time frame. A sterility check was performed regularly by inoculation in peptone-enriched f/2 medium [22]. Four replicates of each treatment were grown in media supplemented with complete f/2 nutrients (control, +P) or phosphate-free f/2 nutrients (-P) in batch cultures with an initial cell density of 5×10^4^ ml^-1^ in 250 ml sterile culture flasks. Cell counting was performed daily using a Bürker-Türk counting chamber. The maximum photosystem II (PSII) quantum yield (F_V_/F_m_) was also measured daily using pulse amplitude modulated (PAM) fluorometry (AquaPen-C AP-C 100, Photon Systems Instruments, Drasov, Czech Republic). Samples were briefly dark-treated (1 min) before measurement of F_m_ (maximum chlorophyll fluorescence) and F_t_ (instantaneous chlorophyll fluorescence, which in dark adapted cells is equivalent to F_o_, emission by excited Chl *a* in PSII antennae). F_v_, the maximum capacity for photosynthetic quenching, was calculated as the difference between F_m_ and F_o_ (F_v_ = F_m_ − F_o_). For the remaining experiments, harvesting was performed 48 h and 72 h after the start of the treatment. Samples for RNA and metabolite analyses were stored at -80°C, whereas samples for element and pigment analyses were kept at -23°C until analysis.

### Nutrient analysis

Samples for particulate nitrogen, carbon and phosphorus analysis were collected on pre-combusted GF/F filters (particulate C and N analysis) or 0.2 μm GF/F filters (particulate phosphorus). Filtrates were used for the analysis of inorganic phosphate and nitrate concentration in the medium. Samples for particulate nitrogen and carbon analysis, along with blank filters, were treated with fuming HCl (37%), packed in tin capsules, dried for two days at 60°C and analyzed by an ECS 4010 CHNSO element analyzer (Costech Instruments, Milan, Italy). NO_3_^-^ + NO_2_^-^ and PO_4_^3-^ were analysed in parallel according to the I.O. Analytical cartridge Parts A002603 and A002604, respectively, as described by Hansen and Koroleff [23]. Particulate phosphate was first oxidized to inorganic PO_4_^3-^ by adding 0.2 g potassium persulphate to 20 ml of culture and boiling at 200 kPa for 30 min according to Norwegian standard NS 4725.

### Pigment analysis

Pigments were analysed following the protocol of Rodriguez et al. [24] on a Hewlett-Packard HPLC 1100 Series system (Hewlett-Packard, Wilmington, IL, USA). Pigments were calculated as femtogram (fg) pigment per cell.

### Neutral lipid measurement

Analysis of neutral lipid content was performed as described in Alipanah et al. [25]. In short, 1 ml of *P*. *tricornutum* cultures were stained with 1 μl of 0.1 μg ml^-1^ BODIPY 505/515 (4,4-Difluoro-1,3,5,7-Tetramethyl-4-Bora-3a-4a-Diaza-s-Indancene, Life Technologies, Carlsbad, CA, USA) and 2% (w/v) dimethyl sulfoxide for 5 min, transferred to a microscope slide, and sealed under a coverslip using dental wax. At least 20 cells for each treatment were analysed for BODIPY 505/515 fluorescence on a TCS SP5 confocal laser-scanning microscope using a 63× water objective (Leica Microsystems, Wetzlar, Germany). Z-sectional images were acquired using an argon laser excitation at 488 nm, and emission was detected with a spectral detector set between 495 nm and 550 nm. A z-stack consisting of ten scans was made for each cell, encompassing the complete fluorescent part of the cell. The length of the z-stack varied between 4.00 μm and 5.78 μm; consequently, the z-slice step size varied between 0.44 μm and 0.64 μm. Image stacks containing the fluorescence channel were imported into ImageJ [26]. The corrected total cell fluorescence for each cell was calculated using the following formula [27, 28]:

Whole-cell signal corrected = whole-cell signal − (number of pixels for the selected cell = surface selected × background).

### Metabolite analysis

Extraction, separation and analysis of metabolites were done as described in Alipanah et al. [25]. Briefly, 60–100 ml of cultures (four replicates) were collected by filtration onto 0.65-μm Durapore membrane filters (Millipore, Billerica, MA, USA), washed off, and finally centrifuged at 16,000 g for 1 min at 4°C. The supernatant was removed and pellets were flash frozen in liquid nitrogen and stored at -80°C. Metabolites were extracted by adding 1 ml of a pre-cooled water:methanol:chloroform (1:2.5:1) mixture containing ribitol (100 μg ml^-1^) as the internal standard. Samples were treated for 60 min at 60μC in an ultrasonic bath, centrifuged for 10 min at 16,000 g, and 600 μl aliquots of supernatants were transferred to 2 ml Eppendorf tubes. Subsequent sample derivatization and GC–MS analysis generally followed the procedures as described in Sissener et al. [29]. Chromatogram visualization and peak identification was performed using Agilent ChemStation software (Agilent Technologies, Waldbronn, Germany), AMDIS software (version 2.71; National Institute of Standards and Technology, Boulder, CO, USA) and OpenChrom Community Edition Synge (version 0.6.0) (Peter Wenig; http://www.openchrom.net). GC–MS data integration, normalization (total signal) and alignment were performed using the MetAlign software (PRI-Rikilt, Wageningen, The Netherlands). Statistical analysis was performed using a one-way ANOVA across all time points and P conditions. Metabolite differences were finally calculated as log2(n) ratios of -P to +P at sampling timepoints 48 h and 72 h (S1 Table).

Hierarchical cluster analysis (HCA) using Pearson correlation (average linkage clustering) was based on mean ratio values (-P vs. +P at 48 and 72 h; n = 4) of 98 metabolites identified both in the N and P experiment. Calculations and heat map visualisation were carried out using the MultiExperiment Viewer software v.4.9.0 [30].

### RNA isolation

Depending on the cell density, 60–100 ml of cultures (four replicates) were collected by filtration onto 0.65 μm Durapore membrane filters, washed off using 1 ml of f/2 medium (-P cells were washed with f/2 without phosphate supplement) and centrifuged at 16,000 g for 1 min at 4°C. The supernatant was removed and pellets were flash frozen in liquid nitrogen and stored at -80°C. RNA extraction, quality and quantification were performed as described in [31]. RNA integrity was checked on a 2100 Bioanalyzer (Agilent Technologies, Waldbronn, Germany). All samples had RNA integrity numbers (RIN) above 7.

### DNA microarray experiments

200 ng of total RNA from three replicates randomly chosen from each treatment was reverse transcribed, amplified and labelled according to the One-Color Low Input Quick Amp Labeling Kit (Agilent; 5190–2305), using Cy3 as labelling dye. A total of 1,650 ng of cRNA from each sample was fragmented and hybridized with Gene Expression Hybridization Kit (Agilent; 5188–5242) on 4×44K *P*. *tricornutum* whole-genome 60-mer oligonucleotide microarrays (Agilent Technologies, Waldbronn, Germany) in an Agilent G2545A Hybridization Rotary Oven at 10 rpm, 65°C for 17.5 hours. Slides were washed with washing buffer 1 and 2 using Gene Expression Wash Buffer Kit (Agilent; 5188–5327) and directly scanned using a laser scanner (G2505 B; Agilent Technologies, Waldbronn, Germany) based on the “dynamic range expander” option in the scanner software. Images were processed by Agilent Feature Extraction software version 9.5.

### Statistical analysis

The Limma (Linear Models for Microarray Data) package (version 3.20.1) [32] and R version 3.0.3 were used for statistical analysis and the identification of significant differentially expressed genes. Single colour feature expression files from the Agilent microarray scans were imported, and spots identified as feature outliers were excluded from the analysis. Weak or non-detected spots were given a reduced weight, using the limma weight function. The data were normalized using the quantile method. Since the background signal was negligible (0.5% of mean spot signal intensity), no background subtraction was performed. A design matrix was created and pair-wise comparisons between the samples (-P 48 h vs +P 48 h, and–P 72 h vs +P 72 h) were performed. The method of Benjamini and Hochberg [33] was used to estimate the false discovery rate. Genes with an adjusted P-value < 0.01 were considered to be significantly differentially expressed and were included in the analysis if all probes for each gene had a mean adjusted P-value < 0.01. The study is Minimum Information About a Microarray Experiment (MIAME) compliant. Raw data have been deposited in GEO (accession GSE66063).

The GO annotation (biological process) was downloaded from the *P*. *tricornutum* database at JGI (http://genome.jgi-psf.org/Phatr2/Phatr2.home.html) and further refined by manual GO curation. Significantly regulated genes (adj. P-value < 0.01) were assigned to the GO terms and listed separately for up- and down-regulated genes at the various time points using a custom made Perl script.

GO terms assigned to significantly regulated genes (adj. P-value < 0.01) from the P deprivation experiment and a previously published N deprivation experiment [25] were listed separately for up- and down-regulated genes as well as for similar, opposite or unique regulation. In total 12 different comparisons were analysed: genes similarly up-regulated in both treatments at 48 and 72 hours, genes similarly down-regulated in both treatments at 48 and 72 hours, genes oppositely regulated at 48 and 72 hours, and unique genes at 48 and 72 hours that were only affected in one of the treatments. The GO analysis was performed using a custom made Perl script.

The DNA microarrays data set was compared with the RNAseq results reported by Yang et al. [34], where a related genome-wide transcriptome analysis related to phosphate stress was reported. Significantly regulated genes (adj. P-value < 0.01) identified in this study, which were found in the RNAseq data set, were grouped into similar or opposite regulated genes using a custom made Perl script. Venn diagrams were created using R and the vennDiagram function included in the Limma Package (version 3.20.1) [32].

### Quantitative real-time PCR

Reverse transcription of 1 μg total RNA from four biological replicates from all treatments was performed with QuantiTect Reverse Transcription kit (Qiagen, Hilden, Germany) following the recommended protocol. Reactions where the reverse transcriptase had been omitted were included for all samples to be used as genomic DNA controls during the quantitative real-time PCR (qRT-PCR) analyses. qRT-PCR reactions were performed on a LightCycler 96 using the LightCycler 480 SYBR Green I Master kit (Roche Applied Science, Mannheim, Germany), with a program including pre-incubation for 10 min at 95°C, followed by 50 cycles of amplification consisting of 10 s at 95°C, 10 s at 55°C and 10 s at 72°C. Primer sequences are provided in S2 Table. The microarray dataset was screened for genes that were non-responsive to P deprivation at both time points. Based on this screen, Phatr2_24186 and Phatr2_28684 were chosen as reference genes for the qRT-PCR analysis. PCR efficiencies and C_t_ values were calculated by linear regression using the LinRegPCR software [35, 36], and the mean PCR efficiency was calculated for each pair of primers. PCR efficiencies and C_t_ values were used in qBASEPlus (Biogazelle, Zwijnaarde, Belgium) software to calculate the statistical significance of the difference in expression levels in various treatments. The target genes were normalized to the reference genes in qBASEPlus.

### Protein sequence analyses

Protein sequences of 6-phosphofructo-2-kinase/fructose-2,6-bisphosphatases (human PFKFB2, Acc. no. O60825; *P*. *tricornutum* PF2K/F2BP1, Acc. no. EEC51177; *P*. *tricornutum* PF2K/F2BP2; Acc. no. EEC51418) were obtained from NCBI. Protein alignment was generated using GeneDoc 2.7.000 [37] and refined using Affinity Designer. The presence of putative signal peptide sequences was investigated using SignalP 4.1 (http://www.cbs.dtu.dk/services/SignalP) [38] and HECTAR (http://webtools.sb-roscoff.fr) [39] prediction servers. Subcellular protein location was predicted using the TargetP (http://www.cbs.dtu.dk/services/TargetP) [38] prediction server.

## Results and discussion

### Physiological and biochemical responses to P limitation

**Fig 1:**
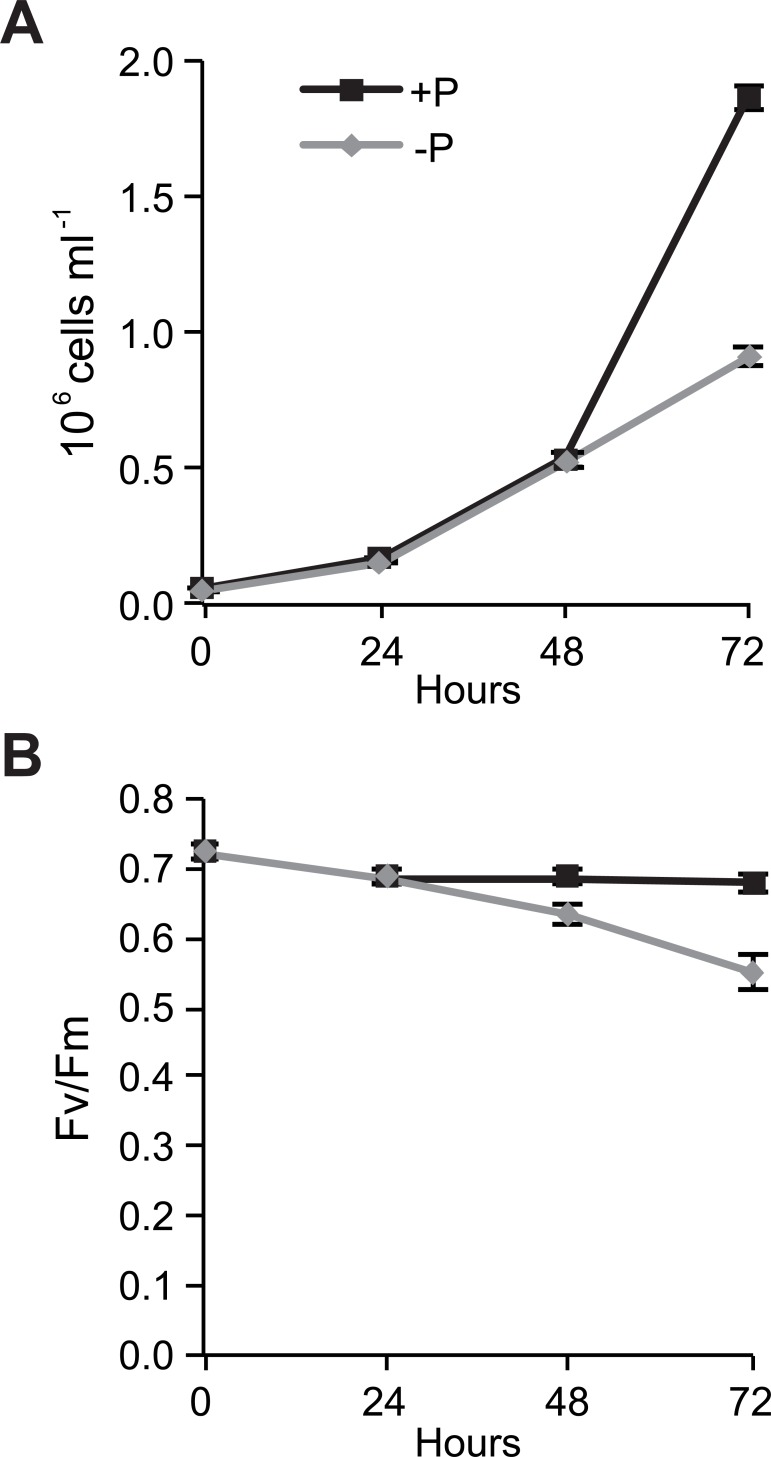
Growth curve and efficiency of PSII in +P and -P cells. (A) Growth of *P*. *tricornutum* in +P (36 μM phosphate) and -P (phosphate-free) cultures. (B) Efficiency of PSII (F_V_/F_m_) in +P and -P cultures from day zero (start of the experiment) to day 3. Values are mean ± 1 SD of four replicates.

*P*. *tricornutum* was cultured in phosphate-replete (+P) and phosphate-free (-P) medium for 72 h. Both -P and +P cultures grew exponentially until 48 h (Fig 1A). Cell growth subsequently decreased in -P cultures and cells entered the stationary growth phase, whereas +P cultures continued their exponential growth. At 72 h, the cell density of +P cultures was more than twice as high as -P cultures. Chlorophyll *a* variable fluorescence (F_v_/F_m_), a proxy for the maximum quantum yield of photosystem II (PSII), was constant (0.68) during the entire experiment in +P cultures (Fig 1B). In -P cultures, F_v_/F_m_ values decreased throughout the experiment, indicating a drop in the photosynthetic capacity. A similar decrease in response to phosphorus deficiency was recently observed in studies on *Thalassiosira weissflogii* and *P*. *tricornutum* [8, 40]. To compare early responses to phosphorus deprivation before and after the limitation was manifested in reduced cell growth, 48 h and 72 h time points were selected for further molecular and biochemical characterization.

We measured the levels of the light-harvesting pigments chlorophyll *a* (Chl *a*) and fucoxanthin and the photoprotective pigments diadinoxanthin and diatoxanthin. -P cultures contained significantly reduced levels of both Chl *a* and fucoxanthin at both time points compared with +P cultures (Table 1). However, the ratio of fucoxanthin to chl *a* was basically unchanged in both treatments. Although chlorophyll molecules do not contain any phosphorus, reduction in photosynthesis levels as a result of reduced P_i_ availability, a prerequisite for the biosynthesis of ATP by ATP synthase, may lead to a lower demand of photon absorption by chlorophylls and ultimately to the suppression of chlorophyll synthesis. A stable fucoxanthin/Chl *a* ratio during nutrient starvation has previously been reported in *P*. *tricornutum* [25, 41], indicating coordinated biosynthesis of these two pigments. However, in P-limited cultures of *T*. *weissflogii* this ratio rapidly declined [40], suggesting that the regulation of light-harvesting pigments in response to nutrients shows substantial interspecies variability, a notion also made by Geider et al. [41]. Diadinoxanthin levels were moderately reduced at both time points in -P cultures. De-epoxidation of diadinoxanthin to diatoxanthin was not detected after 48 h, and found only at low levels in both +P and -P cultures after 72 h, indicating that non-photochemical quenching (NPQ) plays a minor role in the observed reduction of photosynthetic energy conversion efficiency. In contrast, Geider et al. [41] reported a strong conversion of diadinoxanthin to diatoxanthin during P starvation in *P*. *tricornutum*. The observed differences may at least partly be explained by differences in the light conditions, as the experiments by Geider et al. [41] were performed at considerably higher light intensities (250 μmol photons m^-2^ s^-1^) compared to our experiments (60 μmol photons m^-2^ s^-1^).

**Table 1:** Changes in pigment content and chemical composition of phosphorus-replete (+P) and phosphorus-depleted (-P) cultures after 48 h and 72 h. Data are mean ± 1 SD (n = 4). ND, not detected.

	+P 48 h	-P 48 h	+P 72 h	-P 72 h
**Pigment concentration**				
Chl *a*, fg/cell	327.13 ± 13.31	260.10 ± 11.70	344.98 ± 27.38	226.94 ± 12.34
Fucoxanthin, fg/cell	125.91 ± 6.79	96.17 ± 2.27	136.93 ± 9.82	76.08 ± 3.73
Fucoxanthin/Chl *a*	0.38	0.37	0.40	0.34
Diadinoxanthin, fg/cell	208.46 ± 7.38	183.98 ± 16.14	225.03 ± 5.46	189.65 ± 13.20
Diatoxanthin, fg/cell	ND	ND	36.82 ± 11.37	17.65 ± 2.56
**Cellular nutrient content**				
C, pg/cell	11.55 ± 0.63	13.03 ± 0.76	11.55 ± 0.74	14.21 ± 0.81
N, pg/cell	1.92 ± 0.08	1.77 ± 0.11	2.12 ± 0.08	1.48 ± 0.11
P, pg/cell	0.45 ± 0.02	0.13 ± 0.01	0.43 ± 0.03	0.08 ± 0.00

The particulate carbon content increased in -P cells, reaching a level 23% higher than in the +P cells after 72 h (Table 1). In contrast, cellular nitrogen and phosphorus decreased at both time points following P deprivation, and was 70% and 19% of +P levels after 72 h, respectively. Furthermore, metabolite data indicated that intracellular inorganic phosphate was reduced under P deprivation (S1 Table). Phosphate and nitrate concentrations of the medium confirmed that only -P cultures were phosphate deprived (S3 Table).

Cellular neutral lipid accumulation was analysed using the fluorescent dye BODIPY 505/515.

**Fig 2:**
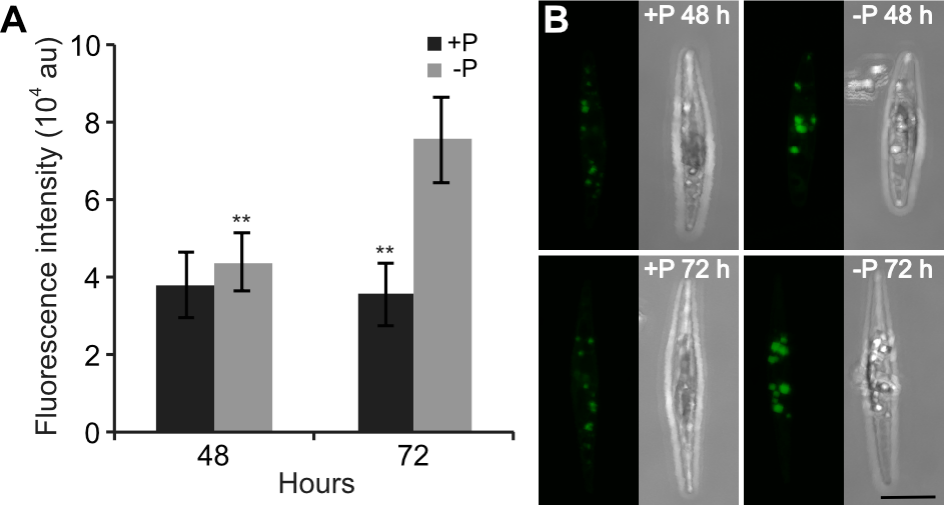
Accumulation of neutral lipids during P deprivation. (A) Fluorescence intensity in *P*. *tricornutum* cells from +P and -P cultures stained with BODIPY 505/515 at 48 h and 72 h. Fluorescence was measured in 20–30 randomly selected cells using confocal microscopy. Significant differences (**, P < 0.01) between +P and -P cultures are indicated. au, arbitrary units. (B) Z-stack projections of *P*. *tricornutum* in +P and -P cultures at 48 h and 72 h. Bar, 5 μm.

A small but significant increase in neutral lipid content was observed at 48 h in -P cells. After 72 h, the neutral lipid levels were two times higher in -P cells than in control cells (Fig 2A). Representative confocal images from the analysis showed the presence of larger and more abundant lipid droplets in -P cells compared with +P cells at both time points (Fig 2B). These results are in line with previous studies on P deprivation in diatoms [14, 34, 42–45]. Accumulation of neutral lipids is part of a general nutrient stress response, in which the metabolism is switched from biomass production to energy storage [46]. In summary, P deprivation leads to inhibition of cell growth, reduction in photosynthetic efficiency and photosynthetic pigments, and increased production of storage lipids.

### Extensive reprogramming of the *P*. *tricornutum* transcriptome in response to P deprivation

A transcriptome analysis was performed on -P and +P samples harvested at 48 h and 72 h using full-genome oligonucleotide microarrays. The treatment led to large transcriptome changes at both time points; with strict statistical filtering (P < 0.01; ≥1.0 ratio filtering), 1,340 and 2,568 transcripts were found to be differentially expressed in -P cultures compared with +P cultures at 48 and 72 h, respectively (S1 Dataset). Six genes displaying strong transcript level responses to P deprivation and encoding proteins involved in different cellular processes were further verified using qRT-PCR, of which the result was consistent with microarray for all six examined genes (S1 Fig). This indicated the differential regulated gene profiles acquired in microarray were credible for the following analysis.

**Fig 3:**
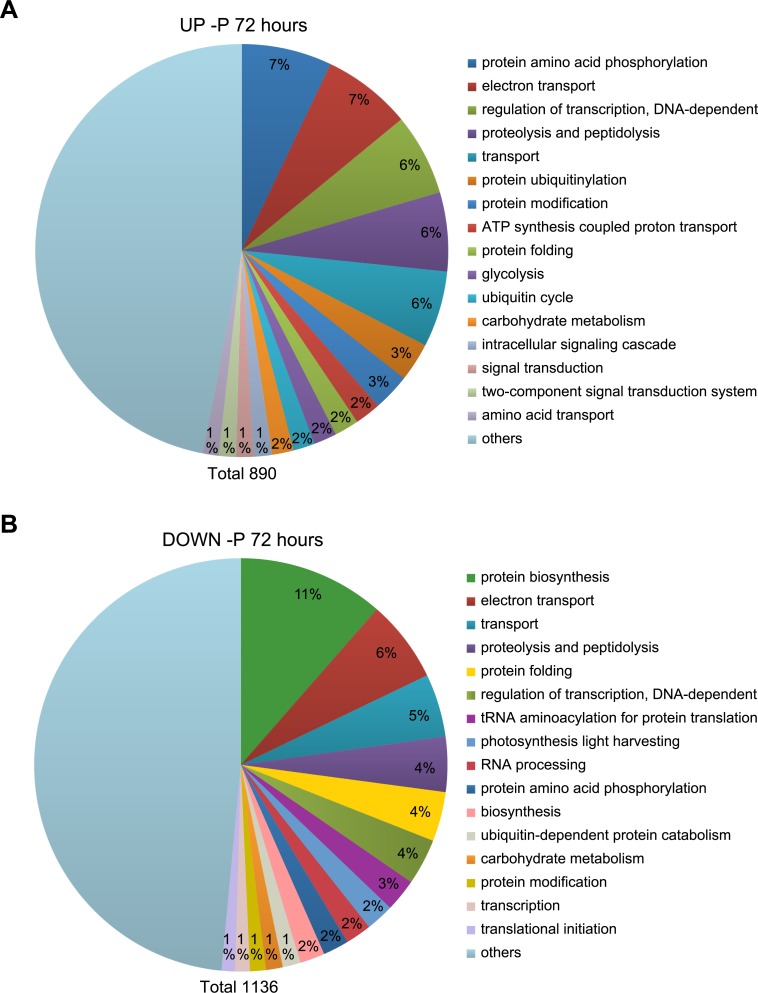
GO analysis of significantly regulated genes after 72 h of P deprivation. The dataset was divided into (A) up- and (B) down-regulated genes and analysed for process GO terms. The 16 most frequent GO terms were listed, and the rest were combined into “others”. The number in the “others” section indicates the percentage of hits within this category. The total number of GO term hits is listed below the diagram.

The transcriptome dataset was subjected to a gene ontology (GO) analysis. The most common GO process terms at 48 h (S2 Fig) and 72 h (Fig 3) were similar, suggesting that the P deprivation response did not change fundamentally from 48 h to 72 h, but rather was enhanced as the deprivation progresses, as indicated by the increased number of regulated transcripts. However, a GO term comparison between up- and down-regulated genes in -P cultures at each time point exhibited large differences. The GO term most frequently assigned to up-regulated genes at 72 h was protein amino acid phosphorylation, which includes both enzymes with phosphorylation and dephosphorylation activities (Fig 3). Signal transduction and ubiquitin-related processes were also up-regulated. The GO term most frequently assigned to down-regulated genes was protein biosynthesis. Genes related to transcription and translation were down-regulated, in support of reduced protein synthesis as a result of reduced cell growth. Genes involved in photosynthesis light harvesting were also down-regulated, in line with the declining photosynthetic capacity of -P cells (Fig 1B).

### Variation of gene regulation between two P deprivation experiments in *P*. *tricornutum*

An RNA-Seq study of P deprivation in *P*. *tricornutum* was recently published [34]. In this study, P deprivation for 48 h was found to lead to the up-regulation of 2,491 and down-regulation of 405 transcripts, with an FDR ≤ 0.001 and log2-fold change ≥1.

**Fig 4:**
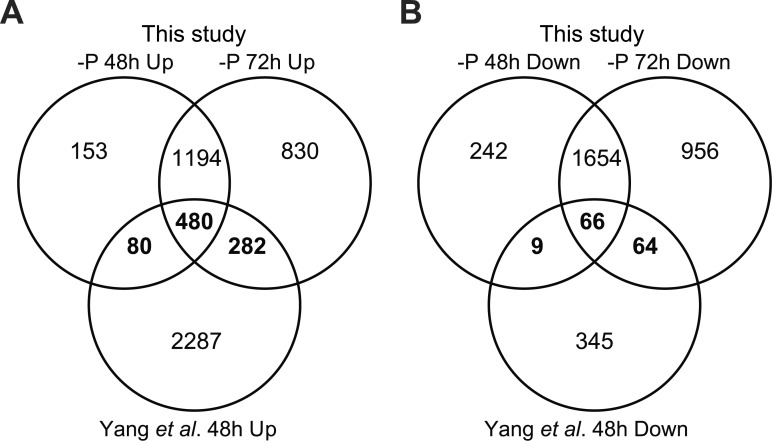
Venn diagram of up- and down-regulated genes from Yang et al. [34] and two microarray datasets (48 h and 72 h) in P-deprived *P*. *tricornutum*. (A) Up-regulated genes. (B) Down-regulated genes. Numbers indicate genes that have shared or unique regulation among the three datasets. Genes similarly regulated in this study and Yang et al. [34] are indicated in bold.

We compared the significantly regulated genes at 48 h and 72 h with the filtered dataset [34]. A majority of the genes were similarly regulated at both time points in our experiment (1,674 up-regulated, Fig 4A; 1,720 down-regulated, Fig 4B). Surprisingly, only 842 of the up-regulated (Fig 4A) and 139 of the down-regulated genes (Fig 4B) in our experiment showed a similar regulation in the Yang et al. [34] dataset. In comparison, 1,241 of the genes that were down-regulated in one or both of the time points in our experiment were up-regulated in the Yang et al. [34] dataset (S3 Fig). A GO analysis of these genes showed an enrichment of terms related to protein synthesis and photosynthesis (S4 Fig).

The large differences between the two datasets are likely caused by the control samples used in Yang et al. [34], which was collected at stationary phase. The cells in the control samples might therefore undergo other stress and induce corresponding regulation of cellular process as response, as supported by the photosynthetic efficiency observed in control group slightly lower that of–P treatment. Thus, the effect of phosphorus deprivation in the -P culture may be difficult to separate from secondary effects of the control culture entering the stationary growth phase in the dataset of Yang et al. [34]. Moreover, the differences between the two datasets could be attributed to strain differences or differences in experimental setups such as cell densities.

### P_i_ acquisition and recycling mechanisms

Up-regulation of P_i_ transporters in response to phosphorus starvation has been observed in microalgae [7, 47]. Our microarray data confirmed that several P_i_ transporters were differentially expressed at 48 h and 72 h, respectively (S1 Dataset). Of the transcripts regulated at 72 h, the mRNA levels of 13 P_i_ transporter-encoding transcripts were up-regulated in -P cells; the transcript level increase of the putative high affinity Pi transporter Phatr2_23830 was also confirmed by qRT-PCR analysis (S1 Fig). P_i_ transporters might be associated with P_i_ acquisition from the intra- or extracellular P_i_ pool. Phatr2_19586, which encodes a protein with similarity (33% identical and 52% similar amino acid residues) to the *Arabidopsis thaliana* VACUOLAR PHOSPHATE TRANSPORTER 1 (VPT1), was highly up-regulated in P-deprived cells. VPT1 functions in vacuolar P_i_ sequestration in *A*. *thaliana* [48]. Therefore, Phatr2_19586 might be responsible for P_i_ transport into the vacuole under phosphorus stress in *P*. *tricornutum*.

The concentration of DOP can exceed that of DIP in coastal and open ocean surface waters, and DOP can be used as an alternative P source [49]. Alkaline phosphatase (APase) is able to break down phosphate from a wide range of intra- and extracellular phosphor-ester compounds, and its activity is induced in *P*. *tricornutum* by P deficiency [9]. Three of four transcripts encoding putative APases or APase-related proteins were among the five most up-regulated genes in our microarray dataset (Phatr2_39432, Phatr2_49678 and Phatr2_45757; S1 Dataset). The induction of three of these transcripts was also observed by Yang [34]. The enhanced expression level of one of the APases (Phatr2_39432) was confirmed through qRT-PCR (S1 Fig). Two of these APases (Phatr2_39432 and Phatr2_45757) belong to the PhoD family, whereas one (Phatr2_49678) contains a PhoA domain. The induction of APase genes is consistent with the up-regulation of several Na^+^/P_i_ transporters, suggesting that *P*. *tricornutum*, like many microalgae, can scavenge P_i_ from DOP when cells become P-limited.

Another interesting feature of *P*. *tricornutum* in response to P deprivation is the up-regulation of 5^′^-nucleotidase enzymes. RNA and DNA are rich sources of P inside the cell, and previous studies have shown that diatoms are able to scavenge P from the nucleotides of degraded RNA or DNA [9]. In our experiment, several 5^′^-nucleotidase transcripts were induced at 72 h (S1 Dataset); the strongest induction was observed for Phatr2_43694 (281 times up-regulated). qRT-PCR analysis confirmed this up-regulation (S1 Fig) Interestingly, Phatr2_43694 carries a putative signal peptide (SignalP [38] score: 0.747; HECTAR [39] signal peptide score: 0.804), indicative of an extracellular localisation. TargetP [38] also predicted Phatr2_43694 to be secreted (TargetP score: 0.968). To our knowledge, no extracellular or cell surface-related 5^′^-nucleotidase has been reported in diatoms. However, marine and freshwater bacteria have a cell surface 5^′^-nucleotidase [50]. In summary, *P*. *tricornutum* employs a variety of P scavenging mechanisms during P deprivation.

### Photosynthesis and nitrogen metabolism

Following P deprivation, the transcript levels of a majority of the genes involved in photosynthetic electron transport and photophosphorylation decreased in *P*. *tricornutum* (S1 Dataset). This was supported by the reduction in F_v_/F_m_ (Fig 1B). Furthermore, transcript level reduction was observed for most of the genes encoding enzymes in the biosynthetic pathways for Chl *a* and accessory pigments (e.g., fucoxanthin), as well as light-harvesting complex (LHC) proteins, implying decreased light energy transport to the photosynthetic apparatus. This is consistent with the reduced Chl *a* and fucoxanthin concentrations (Table 1), and in agreement with previous observations that ratios of light-harvesting pigments are independent of nutrient starvation [41].

Genes associated with nitrate and ammonium assimilation, such as nitrate and ammonium transporters, nitrate reductase (NR), Fd- and NADP nitrite reductases, glutamine synthetase (GSII, Phatr2_51092) and Fd-dependent glutamate synthase (Phatr2_24739) were among the most down-regulated transcripts (S1 Dataset). The down-regulation of GSII was confirmed by qRT-PCR (S1 Fig). The suppression of N uptake might be related to a reduced requirement for protein biosynthesis in -P cells, which was also manifested by strong and concerted downregulation of genes coupled to ribosome biogenesis. Wurch et al. [47] reported that N metabolism was repressed under P deficiency in *A*. *anophagefferens*. P deprivation also affected amino acid metabolism. Several transcripts related to amino acid biosynthesis were repressed, whereas a majority of the genes encoding enzymes involved in the catabolism of amino acids (e.g., branched chain amino acids) were induced. A similar trend was observed in the metabolite data, with reduced levels of amino acids in -P cells (S1 Table). In summary, transcript and metabolite data suggest that both photosynthesis and nitrogen metabolism is repressed during P deprivation.

### Carbon metabolism

**Fig 5:**
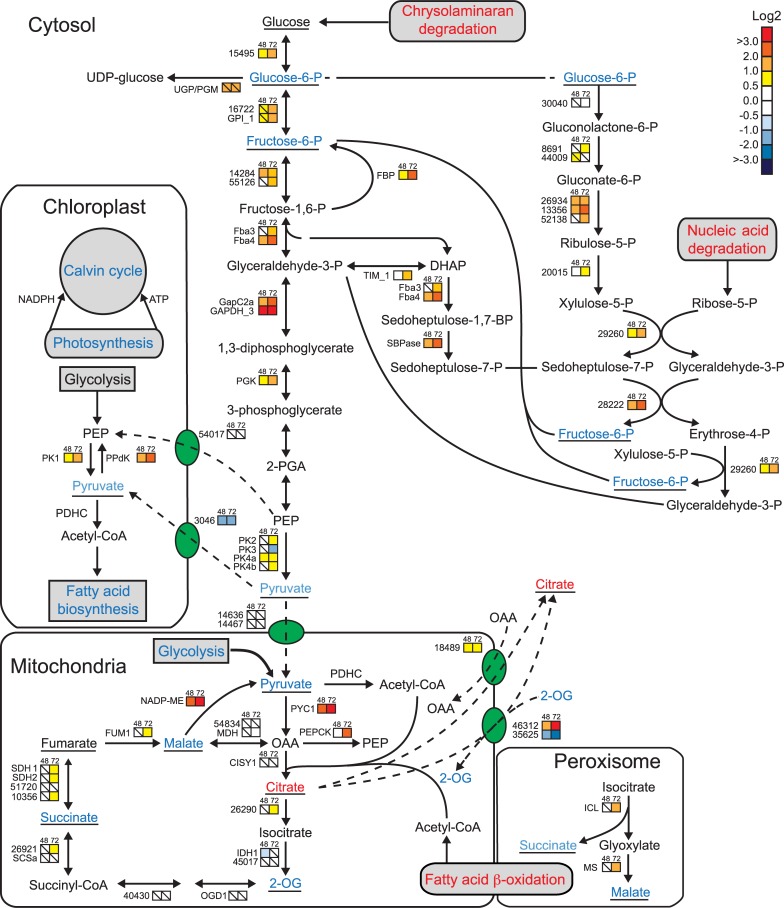
Changes in carbon metabolism in -P cells. Coloured squares indicate the regulation pattern of genes encoding putative enzymes involved in central carbon metabolism after 48 h (left square) and 72 h (right square) of P deprivation, compared with P-replete cultures. Squares with a diagonal line inside indicate no significant difference in expression (P > 0.01). The scale on the right represents gene expression ratio values, log2 transformed. Numbers indicate Phatr2 gene IDs. Metabolites detected are underlined. Red, blue and black text indicates up-, down-, and no regulation of pathways or metabolites, respectively. Red arrow indicates allosteric activation. 2-OG, 2-oxoglutarate; 2-PGA, 2-phosphoglycerate; CISY, citrate synthase; DHAP, dihydroxyacetone phosphate; FBA, fructose-1,6-bisphosphate aldolase; FBP, fructose-1,5-bisphosphatase; FUM, fumarate hydratase; GAPDH, glyceraldehyde-3-phosphate dehydrogenase; GLK, glukokinase; GPI, glucose phosphate isomerase; ICL, isocitrate lyase; IDH, isocitrate dehydrogenase; MDH, malate dehydrogenase; ME, malic enzyme; MS, malate synthase; OAA, oxaloacetic acid; OGD, 2-oxoglutarate dehydrogenase; P2FK/F2BP, 6-phosphofructo-2-kinase/fructose-2,6-bisphosphatase; PDHC, pyruvate dehydrogenase complex; PEP, phosphoenolpyruvate; PEPCK, PEP carboxykinase; PGK, phosphoglycerate kinase; PK, pyruvate kinase; PPDK, pyruvate orthophosphate dikinase; PYC, pyruvate carboxylase; SBP, sedoheptulose bisphosphatase; SCS, succinyl-CoA synthase; SDH, succinate dehydrogenase; TPI, triosephosphate isomerase; UGP/PGM, UDP-glucose pyrophosphorylase/phosphoglucomutase. Annotation for glycolysis/gluconeogenesis enzymes is taken from [52], other annotation is taken from [51].

The down-regulation in transcript levels of several Calvin cycle enzymes at 72 h could be related to a decrease in the photophosphorylation rate and a subsequent drop in the chloroplast ATP and NADPH pool (Fig 5, S1 Dataset). Phosphate deficiency also led to a decline in the protein abundance of both RUBISCO subunits in *A*. *anophagefferens* [13]. Reduced mRNA levels were also observed for a *CA-III* and a plastid beta-carbonic anhydrase (*PtCa1*), which concentrate CO_2_ at the vicinity of RUBISCO.

Despite the decrease in the P_i_ pool and ATP levels, -P cells must generate carbon skeletons and energy for key anabolic processes. Our results indicate that this requirement was at least partly provided through the degradation of chrysolaminarin. The induction of several genes encoding beta-glucosidases and glucanases, as well as glucokinase (GLK, Phatr2_15495), which leads to the conversion to glucose-6-phosphate, was observed (Fig 5). Glucose-6-P synthesized from the chrysolaminarin degradation can be utilized as a precursor by the oxidative pentose phosphate pathway (OPPP) [51]. The mRNA levels of most of the genes related to the OPPP were upregulated (Fig 5). This pathway constitutes an important source of reducing agents in the form of NADPH for biosynthetic processes as well as the production of ribulose-5-phosphate. Thus, the OPPP may be induced during P limitation to compensate for reduced NADPH production through photosynthesis. We also observed an induction in the transcript levels of the putatively cytosolic enzymes required for the reductive part of the pathway. Ribose-5-P, one of the intermediates of this pathway, can be obtained from nucleic acid degradation, which was up-regulated in our experiment (S1 Dataset). Sedoheptulose-7-P was also fed to the pathway via glycolysis/gluconeogenesis (Fig 5).

A majority of the genes involved in cytosolic glycolysis or gluconeogenesis were up-regulated at 48 h and 72 h in -P cells, whereas transcripts encoding components of the TCA cycle showed little regulation (Fig 5, S1 Dataset); these results are in line with previous studies [7, 34, 45, 53]. Most metabolites of glycolysis/gluconeogenesis and the TCA cycle were unchanged or reduced in -P cells. However, citrate levels increased 30-fold and 20-fold at 48 h and 72 h, respectively (Fig 5, S1 Table). Phosphate limitation leads to citrate accumulation in the fungus *Apergillus niger*; it is speculated that export of citrate from mitochondria to the cytosol is important for its accumulation [54]. Two genes encoding mitochondrial citrate shuttles (Phatr2_18489 and Phatr2_46312) are transcriptionally induced in our study (Fig 5, S1 Dataset). High citrate concentration can inhibit phosphofructokinase (PFK), the rate-limiting enzyme of glycolysis [55] while inducing fructose 1,6 bisphosphatase [56], which catalyse the opposite reaction. Interestingly, PFK activity was inhibited by P limitation in the diatom *Achnanthes brevipes* [57]. An important regulatory enzyme of glycolysis is 6-phosphofructo-2-kinase/fructose-2,6-bisphosphatase (PFKFB), which produces fructose-2,6-bisphosphate, a potent allosteric activator of PFK. Crystal structures of human and bovine PFKFB2 show that citrate binds to the 2-kinase domain, occupying the fructose-6-P binding site [58]. A protein alignment of human PFKFB2 and the two PFKPB orthologues in *P*. *tricornutum*, P2FK/F2BP1 (Phatr2_17495) and P2FK/F2BP1 (Phatr2_8706), showed that the 2-kinase domain was well conserved (39% identical and 60% similar amino acid residues between human PFKFB2 and *P*. *tricornutum* P2FK/F2BP1, S6 Fig). Furthermore, all four residues interacting with citrate are conserved between human PFKFB2 and *P*. *tricornutum* PFKFBs. Thus, increased citrate levels may induce a metabolic switch from glycolysis to gluconeogenesis during P deficiency in diatoms, at least in the cytosol. Further biochemical characterisation is needed to confirm this hypothesis.

To initiate gluconeogenesis, oxaloacetic acid (OAA) from the TCA can be converted to phosphoenolpyruvate (PEP) by PEP carboxykinase, which was up-regulated in 72 h -P cells. Two transcripts of the glyoxylate cycle (malate synthase and isocitrate lyase) were also induced at 72 h (Fig 5). Their significant up-regulation might be related to the β-oxidation of fatty acids released from the degradation of membrane lipids. Succinate and malate produced in the glyoxylate cycle can enter the TCA cycle.

Glucose-6-P produced from chrysolaminaran degradation and gluconeogenesis can be converted to UDP-glucose by UGPases. UDP-glucose and other UDP-sugars are required for biosynthesis of extracellular polymeric substances (EPS) secreted into the surrounding environment by microalgae including diatoms [59, 60] under nutrient-limiting conditions, especially phosphorus limitation [61–63]. As a result, transcript induction of a cytosolic UDP-glucose-pyrophosphorylase/phosphoglucomutase (UGP/PGM, Phatr2_50445) could promote EPS production, which acts to excrete surplus carbon skeletons from the cell in -P cells. Taken together, these results suggest that phosphorus deprivation leads to reprogramming of the central carbon metabolism, possibly including a switch in the cytosolic carbon flow from glycolysis to gluconeogenesis.

### Lipid remodelling

Several studies have focused on the changes in the fatty acid content of microalgae under phosphorus stress. Generally, an increase in the total fatty acid content of the cells in P-limited cultures has been observed [42, 64]. However in our study, the transcript levels of genes responsible for *de novo* fatty acid biosynthesis (S1 Dataset) as well as free fatty acids levels (S1 Table) were generally unchanged or down-regulated. Down-regulation of this pathway could be related to the lower cell division rate in -P cells and consequently reduced requirement for fatty acids for membrane lipid synthesis. The discrepancy with other studies, may be attributed to the fact that the duration of our experiment was shorter (3 days) compared with Siron et al. [42] (7 days) and Gong et al. [64] (5 days). Thus, the increase in fatty acid levels could be part of a later response to P deprivation.

**Fig 6:**
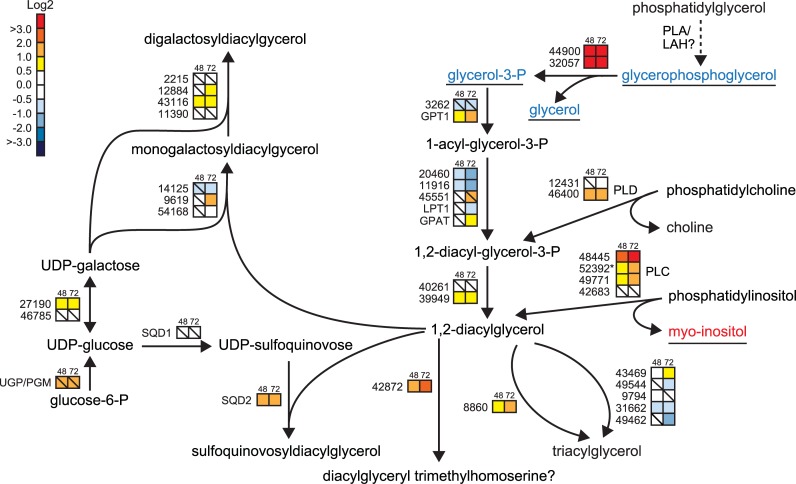
Lipid remodelling in -P cells. Putative enzymes functioning in phospholipid degradation is shown to the right, the triacylglycerol biosynthetic pathway is shown in the middle and non-phosphorus-containing lipid biosynthesis to the left. Coloured squares indicate the regulation pattern of genes after 48 h (left square) and 72 h (right square) of P deprivation, compared with P-replete cultures. Squares with a diagonal line inside indicate no significant difference in expression (P > 0.01). The scale on the right represents gene expression ratio values, log2 transformed. Numbers indicate Phatr2 gene IDs. Metabolites detected are underlined. Red, blue and black text indicates up-, down-, and no regulation of metabolites. The dashed arrow indicates an unidentified enzyme. GPDE, glycerophosphoryldiester phosphodiesterase;; LAH, lipid acyl hydrolase; PLA, phospholipase A; PLC, phospholipase C; PLD, phospholipase D;; UGP/PGM, UDP-glucose pyrophosphorylase/phosphoglucomutase.

Changes in the expression of genes associated with lipid metabolism under P deprivation are shown in Fig 6. Of the eleven phospholipase-encoding genes identified in our microarray data, six were up-regulated at 72 h, three remained unchanged, and two were suppressed (S1 Dataset). Most of the up-regulated phospholipase genes encoded phospholipase C (PLC) and phospholipase D (PLD) isoforms, which lead to the release of diacylglycerol (DAG) and phosphatidic acid (PA), respectively. The induced *PLC* genes all encode phosphoinositide-specific PLCs, and the reaction generates inositol 1,4,5-triphosphate in addition to DAG [65]. The successive dephosphorylation of inositol 1,4,5-triphosphate generates myo-inositol; two genes encoding inositol polyphosphate 5-phosphatases (Phatr2_44344 and Phatr2_43917) were also induced. In support of the PLC-mediated breakdown of phosphatidylinositols, increased levels of *myo*-inositol were observed in -P cells at both time points (S1 Table). Two glycerophosphoryldiester phosphodiesterases (GDPEs, Phatr2_32057 and Phatr2_44900) that may be involved in glycerophospholipid metabolism displayed strongly induced expression by P deprivation; the induction of Phatr2_32057 was confirmed by qRT-PCR analysis (S1 Fig). GDPEs have been shown to catalyse hydrolysis of glycerophosphodiesters to their corresponding alcohols and glycerol-3-phosphate in both bacteria and plants [66, 67]. Interestingly, deacylation of phosphatidylglycerol by a yet unidentified phospholipase A or lipid acyl hydrolase would produce glycerophosphoglycerol, which was found to be strongly reduced in -P cells at both time points (S1 Table).

In P-deficient environments, *T*. *pseudonana* substitutes phosphatidylcholine with the nitrogen-containing betaine lipid diacylglyceryl carboxyhydroxymethylcholine (DGCC) and phosphatidylglycerol with the sulfolipid sulfoquinovosyldiacylglycerol (SQDG), a feature that also has been observed in other phytoplankton [15, 16]. SQDG is synthesized via the two plastidial enzymes UDP-sulfoquinovose synthase (SQD1) and sulfolipid sulfoquinovosyldiacylglycerol synthase (SQD2). *SQD2* expression increased two-fold when P became deprived; no significant change in expression was observed for *SQD1*. This result is supported by observations made in P-limited *T*. *pseudonana* [7]. Furthermore, enhanced levels of the cytosolic UGP/PGM transcript might also contribute to the biosynthesis of sulfolipids (Fig 6).

The replacement of phospholipids with galactolipids is common in plants [68, 69]. Interestingly, up-regulation of one of the genes encoding monogalactosyldiacylglycerol (MGDG) synthase (Phatr2_9619), two putative digalactosyldiacylglycerol (DGDG) synthases (Phatr2_12884, Phatr2_43116; see S1 Dataset), along with reduction in the metabolite level of galactosyl glycerol (S1 Table), was detected at 72 h. A recent glycerolipidome study of *P*. *tricornutum* also showed increased DGDG levels in P-starved cells; the authors postulated that higher content of DGDG compared to decreased amount of MGDG could contribute to sustain the structure of the photosynthetic membrane [44].

*P*. *tricornutum* might channel DAG from the degradation of phospholipids to betaine lipids. It has been proposed that the up-regulation of several methyltransferases might be correlated to betaine lipid synthesis [7]. Recent studies proposed Phatr2_42872 as a candidate gene involved in biosynthesis of the betaine glycerolipid diacylglyceryl-hydroxymethyl-*N*,*N*,*N*-trimethyl-β-alanine (DGTA) [44, 45]. Phatr2_42872 was induced in -P cultures (approximately 5- and 6.5-fold at 48 h and 72 h, respectively) (Fig 6). Interestingly, the expression of this gene was repressed under nitrogen deprivation [25].

P starvation has been shown to result in the accumulation of TAGs in several phytoplankton classes, including diatoms [14, 44, 64]. No general trend in the expression of genes involved in *de novo* TAG biosynthesis could be observed, although some of the genes in the pathway were induced (Fig 6, S1 Dataset). The transcript level of phospholipid:diacylglycerol acyltransferase (PDAT, Phatr2_8860), an enzyme using a phospholipid as an acyl donor for the terminal step of TAG formation, increased 3-fold after 72 h of P deprivation. Phospholipid degradation by PLC and PLD may potentially contribute to TAG accumulation by feeding into the final steps of TAG biosynthesis. In summary, P deprivation-induced lipid remodelling involves several pathways including degradation of different phospholipid classes and biosynthesis of non-phosphorus-containing lipids.

### Comparative transcriptome and metabolite analysis of N and P deprived *P*. *tricornutum*

We recently published a study of transcriptome and metabolite responses to N deprivation in *P*. *tricornutum*, using the same experimental setup as in this study [25]. The transcriptome and metabolite profiles of the N and P deprivation experiments were compared in order to identify similar, opposite and unique (mainly regulated in response to N or P deprivation) regulations.

#### Similar, opposite and unique gene regulation between N- and P-deprived *P*. *tricornutum*

In our comparative analysis, only differentially regulated genes with log2 > 1.0 and P-value < 0.01 were included. Overall, 717 and 1,520 of the differentially regulated genes showed similar regulation between both conditions following 48 h and 72 h of deprivation, respectively (S2 Dataset). A GO analysis of the dataset showed that genes with similar regulation at 48 h were connected to the central carbon metabolism, porphyrin and chlorophyll metabolism, purine/pyrimidine nucleotide biosynthesis, transcription, amino acid biosynthesis, and translation (S4 Table). In addition to processes with similar regulation at 48 h, several genes related to photosynthesis, carotenoid biosynthesis, signal transduction, and fatty acid biosynthesis were also similarly regulated in 72 h N- and P-deprived *P*. *tricornutum*. The changes in these processes appear to be part of a general phosphorus and nitrogen stress response through decrease of cellular metabolism.

Of the differentially expressed genes, 18 (48 h after deprivation) and 59 (72 h after deprivation) were oppositely regulated in -P and -N experiments (S6A Fig, S2 Dataset). The most notable differences were found at 72 h and included genes related to nitrogen uptake and assimilation, sulfolipid biosynthesis, and the betaine lipid synthase-like enzyme (Phatr2_42872).

A variety of transcription patterns were also unique in -P and -N conditions (S2 Dataset). Many genes were also exclusively regulated following P deprivation (48 h, 368 genes; 72 h, 864 genes) (S6B Fig, S4 Table, S2 Dataset). The most up-regulated genes were connected to phosphorus scavenging. Of the P_i_ transporters that were up-regulated in -P cells, six P_i_ transporters were exclusively regulated in P-deprived condition. Ribosomal proteins as well as some transcripts associated with nitrogen transport were among the most down-regulated genes in P-deprived cells.

119 and 370 genes were differentially regulated exclusively in 48 h and 72 h N-deprived cultures, respectively. Genes associated to nitrogen assimilation were among the most up-regulated transcripts in N-deprived cells (S6C Fig, S4 Table, S2 Dataset). The most down-regulated genes were less specific, such as some transcripts connected to photosynthesis, pigment biosynthesis, light harvesting complex, and carbon metabolism.

#### Metabolic changes in *P*. *tricornutum* following N and P deprivation

**Fig 7:**
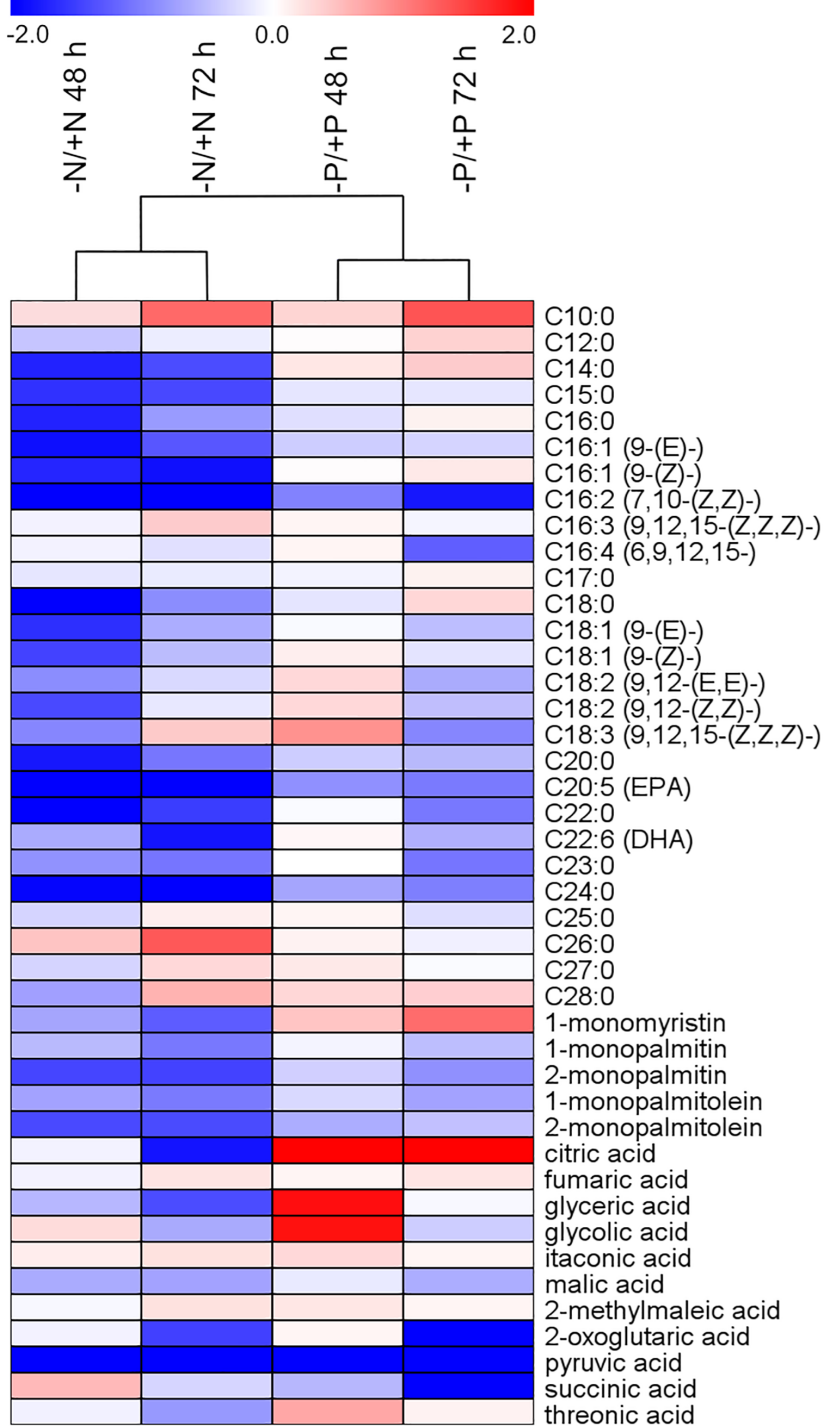
Comparison of the effect of nitrate and phosphate deprivation on fatty acids, glycerides and organic acids. Hierarchical cluster analysis based on mean ratio values, log2 transformed (deprived vs. replete condition at 48 and 72 h; n = 4) from the current P deprivation experiment and a previously published N deprivation experiment [25]. Treatments are depicted in columns while metabolites are represented by rows. Heat map visualization displays differences in metabolite profiles; bluish colours indicate decreased metabolite concentration in deprived compared to the corresponding replete condition, and reddish colours increased metabolite levels (see colour scale).

Fig 7 summarizes the comparative metabolic responses of fatty acids, glycerides and organic acids 48 h and 72 h after N and P deprivation, while S7 Fig shows a comparison of all 112 compounds included in the analysis. Amino acid levels decreased under all conditions, similar to what was observed at transcriptome level, indicating reduced biosynthesis of nitrogenous compounds in both -N and -P cells. Similar metabolic shifts were observed for most of the compounds connected to the central carbon metabolism. However, there was a clear difference in the citrate content of cells following N and P deprivation. While the level of citrate was reduced in -N cells, it strongly increased in -P cells (Fig 7). We propose that citrate accumulation could have an inhibitory effect on glycolysis, switching the carbon flow toward gluconeogenesis in P-deprived cells. In -N cells, the reduction in both pigment levels and efficiency of PSII was stronger [25] compared with -P cells. Geider et al. [41] observed similar responses for PSII efficiency in N- and P-deprived *P*. *tricornutum*. The lower amount of carbon fixed through photosynthesis in -N cells would reduce or eliminate the need to excrete surplus carbon; thus, the carbon flow is still directed through glycolysis to the TCA cycle or fatty acid biosynthesis. In support of this interpretation, a recent transcriptome and proteome analysis of N deprivation in *T*. *pseudonana* indicates that glycolysis is induced while gluconeogenesis is repressed [70]. Furthermore, in a metabolome profiling study of 13 diatom strains during N deprivation, only one of these strains showed a strong increase in citrate levels [71].

Metabolic observations of fatty acids also matched their transcriptional regulation in all deprived conditions. As most components of the fatty acid biosynthetic pathway were transcriptionally down-regulated (S1 Dataset), a majority of the fatty acids showed a decrease in their metabolite levels (Fig 7). However, levels of most medium- to long-chain saturated fatty acids (C12:0 to C18:0) were higher in -P cells, possibly connected to the transcript level increase of genes involved in fatty acid β-oxidation at 72 h (S1 Dataset). The comparative metabolic and transcriptional analyses describe the common and specific processes regulating responses of *P*. *tricornutum* to N and P stress.

### Conclusions

*P*. *tricornutum* was used to elucidate physiological and biochemical adaptations to P deficiency in diatoms. Transcriptional profiles of P-deprived and P-replete cultures at 48 h and 72 h were combined with metabolite profiling and physiological measurements. *P*. *tricornutum* displays extensive modification of metabolic pathways under P deficiency, such as increased import and scavenging of organic and inorganic P and reduced photosynthesis. The central carbon metabolism also displays considerable changes, including induction of the oxidative pentose phosphate pathway and strongly increased levels of citrate, which may act in switching the cytosolic carbon flow from glycolysis toward gluconeogenesis. An analysis of lipid metabolism provides possible mechanisms for the substitution of phospholipids with non-phosphorus lipids and the observed accumulation of TAGs in P-deprived cultures. Finally, a comparative analysis of N- and P-deprived *P*. *tricornutum* highlights similar, opposite and unique regulation patterns of both genes and metabolites. This study provides valuable information on diatom responses to P deprivation, and also enables the separation of P-specific and general nutrient stress responses.

## Supporting information

S1 Fig qRT-PCR analysis of gene responses to phosphorus deprivation in *P*. *tricornutum*. Bars represent relative gene expression values (log2 transformed) in–P cultures compared to +P cultures for microarray (n = 3) and qRT-PCR analyses (n = 4). Numbers indicate JGI Phatr2 gene IDs. All expression ratios analysed by qRT-PCR were statistically significant (P < 0.01). *The expression level of Phatr2_39432 in +P cultures was below detection limit in two replicates, both at 48 h and 72 h. 39432, alkaline phosphatase; 23830, high affinity phosphate transporter; 32057, glycerophosphoryl diester phosphodiesterase; 43694, 5'-nucleotidase; PYC2, pyruvate decarboxylase; GSII, glutamine synthetase.
(EPS)

S2 Fig GO analysis of significantly regulated genes after 48 h of phosphate deprivation. The dataset was divided into (A) up- and (B) down-regulated genes and analysed for process GO terms. The 16 most common GO terms are listed, and the rest are referred to as “others”. The number in each section indicates the percentage of hits within that category, and the total number of GO term hits is listed below the diagram.
(EPS)

S3 Fig Venn diagram of oppositely regulated genes in P-deprived *P*. *tricornutum* from Yang *et al*. [34] and this study (48 h and 72 h). (A) Genes down-regulated in this study and up-regulated in Yang et al. (B) Genes up-regulated in this study and down-regulated in Yang et al. Numbers indicate genes that have shared or unique regulation among the three datasets.
(EPS)

S4 Fig GO analysis of oppositely regulated genes in P-deprived *P*. *tricornutum* from Yang et al. [34] and this study. (A) Genes down-regulated after 48 h in this study and up-regulated in Yang et al. (B) Genes down-regulated after 48 h in this study and up-regulated in Yang et al. The 16 most common GO process terms are shown and the rest are referred to as “others”. The number in each section indicates the number of hits within that category.
(EPS)

S5 Fig Protein alignment of human and *P*. *tricornutum* 6-phosphofructo-2-kinase/fructose-2,6-bisphosphatases. Human PFKFB2 (GenBank Acc. no. O60825) and *P*. *tricornutum* PF2K/F2BP1 (Phatr2_17495, GenBank Acc. no. EEC51177) and PF2K/F2BP2 (Phatr2_8706, GenBank Acc. no. EEC51418). Asterisks indicate residues in the human PFKFB2 2-kinase domain interacting with citrate [58]. The red and blue bars indicated the kinase and phosphatase domains, respectively. The PtPF2K/F2BP2 protein sequence was partial, and its coding sequence was extended at the 3’- and 5’-end. The modified coding sequence was supported by ESTs.
(PDF)

S6 Fig Genes differentially expressed during N and P deprivation in *P*. *tricornutum*. (A) Oppositely regulated genes. (B) Genes uniquely regulated by P deprivation. (C) Genes uniquely regulated by N deprivation. The ratios are log2 transformed. Numbers indicate Phatr2 gene IDs. Gene description is provided below the panel.
(EPS)

S7 Fig Hierarchical cluster analysis between N- and P-deprived treatments. Hierarchical cluster analysis based on mean ratio values, log2 transformed (deprived vs. replete condition at 48 and 72 h; n = 4) from the N- and P-experiment. Treatments are depicted in columns while metabolites are represented by rows. Heat map visualization displays differences in metabolite profiles; bluish colours indicate decreased metabolite concentration in deprived compared to the corresponding replete condition, and reddish colours increased metabolite levels (see colour scale).
(TIFF)

S1 Table Tentatively identified algal metabolites based on GC-MS profiling. (DOCX)

S2 Table Genes analysed by qRT-PCR and their respective primers. (DOCX)

S3 Table Concentration of nitrate and phosphate in the medium of +P and -P cultures. (DOCX)

S4 Table GO analysis between N- and P-deprived treatments. (DOCX)

S1 Dataset Transcriptional responses to P deficiency in *P*. *tricornutum*. (XLSX)

S2 Dataset Comparative transcriptome analysis of N deprivation (Alipanah et al., 2105) and P deprivation (this study). (XLS)
