# Supplementary material for: Molecular adaptations to phosphorus deprivation and comparison with nitrogen deprivation responses in the diatom Phaeodactylum tricornutum
Source: PLoS One. 2018 Feb 23;13(2):e0193335. doi: 10.1371/journal.pone.0193335 (PMC5825098; doi:10.1371/journal.pone.0193335)
Supplement: S5 Fig — Human PFKFB2 (GenBank Acc. no. O60825) and P. tricornutum PF2K/F2BP1 (Phatr2_17495, GenBank Acc. no. EEC51177) and PF2K/F2BP2 (Phatr2_8706, GenBank Acc. no. EEC51418). Asterisks indicate residues in the human PFKFB2 2-kinase domain interacting with citrate [58]. The red and blue bars indicated the kinase and phosphatase domains, respectively. The PtPF2K/F2BP2 protein sequence was partial, and its coding sequence was extended at the 3’- and 5’-end. The modified coding sequence was supported by ESTs. (PDF) [file pone.0193335.s005.pdf]

```
HsPFKFB2     : MSGASSSEQNNNSYETKTPNLRMSEKKCSWASYMTNSPTL-------------------- :  40
PtP2FK/F2BP1 : MSSLNSIPASSSVSDLLSSQRFRPPPNIDNPMPHRLPEGENAAPLEVTGTTASDKMV--- :  57
PtP2FK/F2BP2 : MSSIHSPPSVNGRDHFQYHAEESPPPPPSGLPPTSPTVRKDYYAAGSSELATETPVTVQR :  60

HsPFKFB2     : ---------------IVMIGLPARGKTYVSKKLTRYLNWIGV-PTKVFNLGVYRREAVKS :  84
PtP2FK/F2BP1 : ---------------IVMVGLPATGKTHIAKRICRFLSFFHDIPSQIFNVGDYRRQLCGA : 102
PtP2FK/F2BP2 : LEARRKLTKNSTRLVIALVGLPARGKSFVARKLLHYLNWSGV-QCKIFNVGRYRREAYKH : 119

HsPFKFB2     : YKSY-------------DFFRHDNEEAMKIRKQCALVALEDVKAYLTEENGQIAVFDATN : 131
PtP2FK/F2BP1 : QMPA-------------DFYDPSNEAGQLARHQACDAALADLLEYMKQDGVRVAAFDATN : 149
PtP2FK/F2BP2 : VAAASADARAQTGACDADFFDAQNERAAELREKVADLALRDMLRCFSNHER-IAIFDATN : 178

HsPFKFB2     : TTRERRDMILNFAEQNSFK------VFFVESVCDDPDVIAANILEVKVSSPDYPERNREN : 185
PtP2FK/F2BP1 : STKARRSHILQVLKASGLGCK----RMFVESVCDHEALLEENIRKVKLSTPDYRDMDPEQ : 205
PtP2FK/F2BP2 : STDKRRKWLLQECTSPDKRPGKPTGVVFVESICDDQELLEENYRYKISNSPDFDGMTQQE : 238

HsPFKFB2     : VMEDFLKRIECYKVTYRPLDPDNYDKDLSFIKVINVGQRFLVNRVQDYIQSKIVYYLMNI : 245
PtP2FK/F2BP1 : AMRDFKTRRENYMRVYEPVD----ETDGPHIK-IINSKQFIVTNIRGYLPLKVVHYVMNL : 260
PtP2FK/F2BP2 : ALSDLRKRVTKYEEQYETIT----DDSLSYMKVFNLSTKLMVNHIYGRMAKELVPALMSW : 294

HsPFKFB2     : HVQPRTIYLCRXGESEFNLLGKIGGDSGLSVRGKQFAQALRKFLEEQEITDLKVWTSQLK : 305
PtP2FK/F2BP1 : HTLPRTFYLTRHGQSEYNLLGKIGGDSGLTPAGLEYARRLAQFAQTHIGSQTVTNETTGE : 320
PtP2FK/F2BP2 : HIGTRPVFLCRPGQTISGILTDGEDYVARNKANHENEGPRDPFPMKIITSTMPRAADTVN : 354

HsPFKFB2     : RTIQTAESLGVPYEQ----------------------------------WKILNEIDAGV : 331
PtP2FK/F2BP1 : SATVPRPARLWTSTLRRTLETAQFINHEALQHTWDNGDSAEWLQFRPMARRNLDELYAGT : 380
PtP2FK/F2BP2 : WTDYEFAIQQMS---------------------------------------NLNPLDKGD : 375

HsPFKFB2     : CEEMTYAEIEKRYPEEFALRDQEKYL--YRYPGGESYQDLVQRLEPVIMELERQGNV-LV : 388
PtP2FK/F2BP1 : CDGMTYKEIEQVYPEEFARRQDDKLS--YRYPRGESYMDVTLRLEPLAQEMERTREPVLI : 438
PtP2FK/F2BP2 : FAGMELDEIRKKNPSWYERLERNPYQTRYVFPGGESYADLVKRLTSVVIDVEQQVTPTLV : 435

HsPFKFB2     : ISHQAVMRCLLAYFLDKGADELPYLRCPLHTIFKLTPVAYGCKVETIKLNVEAVNTHRDK : 448
PtP2FK/F2BP1 : VGHQGILRILYAYFMGLDRNEAPYVSIPLNNVIELTPHAYGCHEKRFCLMRKEEMLNDGQ : 498
PtP2FK/F2BP2 : VSHVSILQCLMSYFRNTPVELCTGIEVPMHTVVKFTPVRGGGWSETHHPLFGADEEGKGM : 495

HsPFKFB2     : PTNNFPKNQTPVRMRRNSFTPLSSSNTIRRPRNYSVGSRPLKPLSPLRAQDMQEGAD : 505
PtP2FK/F2BP1 : DEPVTSMPVKTNGGTSHREPGASTVRPAYYAANDPVMNAPSC--------------- : 540
PtP2FK/F2BP2 : I-PVASESEFSQVTFSGSDSPIWTDLVSSKSSSSLKRELSGSKQGSHATS------- : 544
```
