# Supplementary figures and images for: Molecular adaptations to phosphorus deprivation and comparison with nitrogen deprivation responses in the diatom Phaeodactylum tricornutum
Source: PLoS One. 2018 Feb 23;13(2):e0193335. doi: 10.1371/journal.pone.0193335 (PMC5825098; doi:10.1371/journal.pone.0193335)

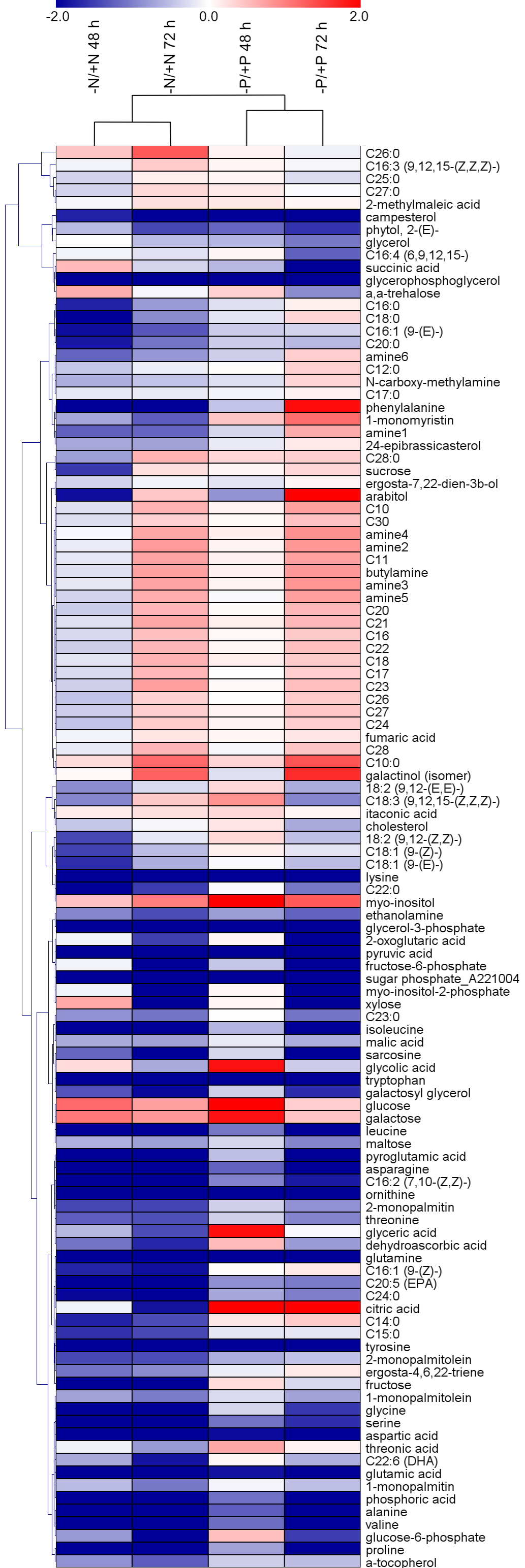

Supplement: S7 Fig — Hierarchical cluster analysis based on mean ratio values, log2 transformed (deprived vs. replete condition at 48 and 72 h; n = 4) from the N- and P-experiment. Treatments are depicted in columns while metabolites are represented by rows. Heat map visualization displays differences in metabolite profiles; bluish colours indicate decreased metabolite concentration in deprived compared to the corresponding replete condition, and reddish colours increased metabolite levels (see colour scale). (TIFF) [file pone.0193335.s007.tiff]
