# Supplementary material for: Molecular adaptations to phosphorus deprivation and comparison with nitrogen deprivation responses in the diatom Phaeodactylum tricornutum
Source: PLoS One. 2018 Feb 23;13(2):e0193335. doi: 10.1371/journal.pone.0193335 (PMC5825098; doi:10.1371/journal.pone.0193335)
Supplement: S1 Table — (DOCX) [file pone.0193335.s008.docx]

**S1 Table.** Tentatively identified algal metabolites based on GC–MS profiling. Values represent log2(n) ratios of P-deprived (-P) to P replete (+P) cultures (n = 4) at sampling timepoints 48 h and 72 h. Colour codes indicate concentration fold changes: increase - ◼ 1.20-1.40, ◼ 1.40-1.67, ◼ 1.67-2.00, ◼ >2 fold; decrease - ◼ 1.20-1.40, ◼ 1.40-1.67, ◼ 1.67-2.00, ◼ >2 fold. P-value was calculated using one-way ANOVA.

| **COMPOUND GROUP** | **METABOLITE** | **ratio -P/+P 48 h** | **ratio -P/+P 72 h** | ***p*-value** |
| --- | --- | --- | --- | --- |
| **Alkanes** | C10 | 0.1061 | 0.7500 | *0.0001* |
|  | C11 | 0.1104 | 0.7449 | *0.0001* |
|  | C16 | 0.0558 | 0.4261 | *0.0017* |
|  | C17 | 0.0088 | 0.3766 | *0.0014* |
|  | C18 | 0.1389 | 0.4023 | *0.0010* |
|  | C20 | 0.0423 | 0.5635 | *0.0001* |
|  | C21 | 0.1408 | 0.5578 | *0.0000* |
|  | C22 | 0.0757 | 0.4970 | *0.0002* |
|  | C23 | 0.0533 | 0.5081 | *0.0000* |
|  | C24 | 0.1036 | 0.3948 | *0.0003* |
|  | C26 | 0.0149 | 0.4079 | *0.0003* |
|  | C27 | 0.1029 | 0.4515 | *0.0004* |
|  | C28 | -0.0636 | 0.4580 | *0.0061* |
|  | C30 | 0.0409 | 0.4813 | *0.0193* |
| **Amines** | butylamine | 0.1116 | 0.8087 | *0.0000* |
|  | N-carboxy-methylamine | -0.2455 | 0.3300 | *0.8691* |
|  | *amine1* | -0.3142 | 0.6715 | *0.0011* |
|  | *amine2* | 0.1053 | 0.8215 | *0.0000* |
|  | *amine3* | 0.1037 | 0.8433 | *0.0000* |
|  | *amine4* | 0.1529 | 0.8706 | *0.0000* |
|  | *amine5* | -0.0446 | 0.7539 | *0.0004* |
|  | ethanolamine | -0.7698 | -1.2239 | *0.0000* |
|  | *amine6* | -0.3823 | 0.3939 | *0.0009* |
| **Amino Acids** | alanine | -1.2567 | -2.9113 | *0.0000* |
|  | sarcosine | -0.3212 | -2.1127 | *0.0003* |
|  | *amino acid1* | 0.1497 | 0.6340 | *0.0032* |
|  | *glycine derivate* | -1.6984 | -3.5714 | *0.0001* |
|  | valine | -1.1435 | -3.0184 | *0.0000* |
|  | *amino acid2* | 0.7382 | 2.4715 | *0.0000* |
|  | leucine | -1.0612 | -3.6100 | *0.0000* |
|  | isoleucine | -0.5810 | -3.0246 | *0.0000* |
|  | proline | -0.7221 | -2.8923 | *0.0000* |
|  | glycine | -0.3342 | -1.5645 | *0.0000* |
|  | serine | -1.0892 | -1.6426 | *0.0000* |
|  | threonine | -0.3632 | -0.9533 | *0.0000* |
|  | pyroglutamic acid | -0.5096 | -2.3843 | *0.0000* |
|  | aspartic acid | -1.9214 | -4.8125 | *0.0000* |
|  | asparagine | -1.2289 | -2.2831 | *0.0019* |
|  | glutamic acid | -1.8565 | -4.0457 | *0.0000* |
|  | phenylalanine | -0.4632 | 1.9199 | *0.0000* |
|  | *amino acid3* | -0.4345 | 0.3322 | *0.0000* |

*Table S1 - continued*

| **Amino acids** | glutamine | -2.6788 | -2.5850 | *0.0173* |
| --- | --- | --- | --- | --- |
|  | ornithine | -2.5147 | -4.4666 | *0.0000* |
|  | lysine | -2.8916 | -3.7953 | *0.0000* |
|  | tyrosine | -2.9263 | -2.7480 | *0.0005* |
|  | tryptophan | -3.2974 | -4.4216 | *0.0068* |
| **Fatty Acids** | C7:0 | 0.1758 | 0.9439 | *0.0000* |
|  | C10:0 | 0.3398 | 1.3481 | *0.0018* |
|  | C12:0 | 0.0291 | 0.3756 | *0.6112* |
|  | C14:0 | 0.1985 | 0.4222 | *0.0956* |
|  | C15:0 | -0.1990 | -0.1977 | *0.2238* |
|  | C16:4 (6,9,12,15-) | 0.0845 | -1.2430 | *0.0274* |
|  | C16:3 (9,12,15-(*Z,Z,Z*)-) | 0.0845 | -0.0702 | *0.0004* |
|  | C16:1 (9-(*E*)-) | -0.3960 | -0.3439 | *0.0406* |
|  | C16:2 (7,10-(*Z,Z*)-) | -0.9778 | -1.8056 | *0.0004* |
|  | C16:1 (9-(*Z*)-) | 0.0273 | 0.1828 | *0.1618* |
|  | C16:0 | -0.2450 | 0.1247 | *0.0104* |
|  | C17:0 | -0.0925 | 0.1132 | *0.9198* |
|  | *fatty acid deriv1 (C18)* | 1.6661 | 1.0438 | *0.0169* |
|  | 18:2 (9,12-(*Z,Z*)-) | 0.3081 | -0.4895 | *0.5812* |
|  | C18:1 (9-(*Z*)-) | 0.1322 | -0.2193 | *0.8013* |
|  | C18:3 (9,12,15-(*Z,Z,Z*)-) | 0.8638 | -0.9412 | *0.4513* |
|  | 18:2 (9,12-(*E,E*)-) | 0.2995 | -0.6529 | *0.6365* |
|  | C18:1 (9-(*E*)-) | -0.0416 | -0.5138 | *0.7678* |
|  | C18:0 | -0.2096 | 0.3291 | *0.7261* |
|  | C20:5 (EPA) | -0.8778 | -1.0416 | *0.0081* |
|  | C20:0 | -0.3967 | -0.5485 | *0.5189* |
|  | C22:6 (DHA) | 0.0638 | -0.6126 | *0.7706* |
|  | C22:0 | -0.0247 | -1.0657 | *0.2309* |
|  | C23:0 | 0.0134 | -1.0887 | *0.3921* |
|  | C24:0 | -0.6975 | -1.0066 | *0.0603* |
|  | C25:0 | 0.0845 | -0.2614 | *0.6497* |
|  | C26:0 | 0.1034 | -0.1154 | *0.0043* |
|  | C27:0 | 0.1755 | -0.0324 | *0.2749* |
|  | C28:0 | 0.3178 | 0.3775 | *0.3708* |
| **Glycerides** | 1-monomyristin | 0.4578 | 1.1533 | *0.0037* |
|  | 2-monopalmitolein | -0.6382 | -0.4727 | *0.0047* |
|  | 2-monopalmitin | -0.3739 | -0.8646 | *0.0346* |
|  | 1-monopalmitolein | -0.2950 | -0.7186 | *0.0242* |
|  | 1-monopalmitin | -0.0793 | -0.5033 | *0.2143* |
| **Organic Acids** | pyruvic acid | -2.2902 | -3.7922 | *0.0000* |
|  | glycolic acid | 1.8773 | -0.3997 | *0.0015* |
|  | succinic acid | -0.5549 | -2.7410 | *0.0000* |
|  | glyceric acid | 1.8950 | -0.0525 | *0.0647* |
|  | itaconic acid | 0.2990 | 0.0794 | *0.0065* |
|  | fumaric acid | 0.0845 | 0.2051 | *0.0003* |
|  | 2-methylmaleic acid | 0.1890 | 0.0794 | *0.0002* |
|  | malic acid | -0.1674 | -0.6399 | *0.0066* |

*Table S1 - continued*

| **Organic Acids** | threonic acid | 0.7025 | 0.1052 | *0.0024* |
| --- | --- | --- | --- | --- |
|  | 2-oxoglutaric acid | 0.0845 | -2.0715 | *0.0013* |
|  | 3-deoxy-D-ribo-hexonic acid | 1.0691 | -0.3546 | *0.1128* |
|  | citric acid | 5.0484 | 3.7734 | *0.0001* |
|  | dehydroascorbic ascorbic | 0.5479 | -0.7807 | *0.1192* |
| **Phosphates** | phosphoric acid | -1.1181 | -2.3596 | *0.0001* |
|  | glycerol-3-phosphate | -3.2449 | -5.8076 | *0.0000* |
|  | glycerophosphoglycerol | -4.3862 | -7.0057 | *0.0000* |
|  | *sugar phosphate_A221004* | -2.3180 | -3.9204 | *0.0001* |
|  | fructose-6-phosphate | -0.4503 | -3.3860 | *0.0000* |
|  | glucose-6-phosphate | 0.4948 | -1.5143 | *0.0188* |
|  | *myo*-inositol-2-phosphate | 0.0845 | -2.7909 | *0.0000* |
| **Sterols & Terpenes** | phytol, 2-(*E*)- | -1.1997 | -1.5934 | *0.0000* |
|  | ergosta-4,6,22-triene | -0.1336 | 0.1801 | *0.0191* |
|  | 24-epibrassicasterol | -0.1658 | 0.1807 | *0.2124* |
|  | cholesterol | 0.2100 | -0.6686 | *0.2091* |
|  | ergosta-​7,​22-​dien-​3β-​ol | -0.2191 | 0.0821 | *0.0256* |
|  | campesterol | -3.0366 | -3.1146 | *0.0000* |
| **Sugars & Polyols** | glycerol | -0.5845 | -1.0624 | *0.0000* |
|  | xylose | 0.0845 | -3.2766 | *0.0001* |
|  | arabitol | -0.8393 | 2.1050 | *0.0169* |
|  | fructose | 0.2687 | -0.3049 | *0.1244* |
|  | glucose | 2.1071 | 0.3980 | *0.0001* |
|  | galactose | 1.8708 | 0.4751 | *0.0000* |
|  | *myo*-inositol | 2.2525 | 1.3088 | *0.0000* |
|  | galactosyl glycerol | -0.3744 | -1.6491 | *0.0030* |
|  | sucrose | 0.0845 | 0.3142 | *0.0055* |
|  | *disaccharide1* | 0.0845 | 0.0794 | *0.0000* |
|  | α,α-trehalose | 0.3698 | -0.9055 | *0.0009* |
|  | *disaccharide2* | 0.0428 | 0.3200 | *0.0056* |
|  | maltose | -0.3179 | -0.9537 | *0.0001* |
|  | *disaccharide3* | -0.8750 | -1.0088 | *0.0033* |
|  | galactinol (isomer) | -0.2485 | 1.6597 | *0.0032* |
|  | *trisaccharide1* | -0.8114 | -1.5050 | *0.0005* |
|  | *trisaccharide2* | -0.8318 | -2.5407 | *0.0321* |
| **Others** | NA_A153004 | 0.7061 | 0.1277 | *0.0372* |
|  | NA_A166004 | -2.0233 | -3.8438 | *0.0004* |
|  | NA_A172005 | -2.1720 | -4.2681 | *0.0193* |
|  | NA_A180013 | -1.8327 | -0.9134 | *0.1085* |
|  | NA_A203005 | 1.0299 | -0.6574 | *0.0136* |
|  | α-tocopherol | -0.3780 | -0.5132 | *0.1104* |
