# Supplementary material for: Molecular adaptations to phosphorus deprivation and comparison with nitrogen deprivation responses in the diatom Phaeodactylum tricornutum
Source: PLoS One. 2018 Feb 23;13(2):e0193335. doi: 10.1371/journal.pone.0193335 (PMC5825098; doi:10.1371/journal.pone.0193335)
Supplement: S2 Table — (DOCX) [file pone.0193335.s009.docx]

**S2 Table.** Genes analysed by real time qPCR and their respective primers.

| JGI Gene ID | Accession | Description | Orientation | Sequence | Amplicon size (bp) |
| --- | --- | --- | --- | --- | --- |
| Phatr2_39432 | XM_002183333 | PhoD-like phosphatase | forward | TGGCCACCATAGCTTCCTTTGA | 107 |
|  |  |  | reverse | ATTTGTGTTCACCGCACCTACA |  |
| Phatr2_23830 | XM_002184974 | Putatively secreted phosphate transporter | forward | TGACAAACTCGAGCTGTACTGG | 128 |
|  |  |  | reverse | CGGCTGTTTGACGCTTTGCGAT |  |
| Phatr2_32057 | XM_002177287 | Glycerophosphoryl diester phosphodiesterase | forward | TTTGCCAACTGTATGGGTGCAT | 67 |
|  |  |  | reverse | TTACTGCCTTCAACGGCAACAT |  |
| Phatr2_51092 | XM_002182173 | Glutamine synthetase (GSII) | forward | GAAGGTAACGAACTTCGCCTGA | 71 |
|  |  |  | reverse | GGCAACACCGTAGCAAAACTTG |  |
| Phatr2_49339 | XM_002183870 | Pyruvate carboxylase 2 (PYC2) | forward | GTGGAACTCGTTTCTATCCAAG | 116 |
|  |  |  | reverse | CGAATCTCCTAACAAGTTCTGG |  |
| Phatr2_24186 | XM_002185447 | Exportin 1-like protein (XPO1) | forward | TCTATTGTTTGGGCGATGAAGC | 89 |
|  |  |  | reverse | CTTACCGACATTAACCAGCAGT |  |
| Phatr2_28684 | XM_002181523 | u4 tri-snrnp-associated 65 kda protein | forward | AACCTTGATCTTGGCGAGTACG | 100 |
|  |  |  | reverse | GCAACCAAATCGTACTTCTGAC |  |
