# Supplementary material for: Molecular adaptations to phosphorus deprivation and comparison with nitrogen deprivation responses in the diatom Phaeodactylum tricornutum
Source: PLoS One. 2018 Feb 23;13(2):e0193335. doi: 10.1371/journal.pone.0193335 (PMC5825098; doi:10.1371/journal.pone.0193335)
Supplement: S3 Table — (DOCX) [file pone.0193335.s010.docx]

**S3 Table.** Time course changes in the medium concentration of nitrate (DIN, NO_3_^-^ + NO_2_) and phosphate (DIP, PO_4_^3-^) in +P and -P cultures 48 and 72 h after P deprivation. Values are means ±SD of four biological replicates.

| **Culture** | **µg PO_4_^3-^ /l** | **µg (NO_3_^-^+NO_2_^-^) /l** |
| --- | --- | --- |
| +P 48 h | 612.77 ± 2.39 | 8797.38 ± 49.23 |
| –P 48 h | 14.28 ± 0.64 | 8929.78 ± 32.32 |
| +P 72 h | 316.99 ± 26.73 | 7550.87 ± 108.79 |
| –P 72 h | 14.00 ± 0.77 | 8799.05 ± 61.49 |
