# Supplementary material for: Molecular adaptations to phosphorus deprivation and comparison with nitrogen deprivation responses in the diatom Phaeodactylum tricornutum
Source: PLoS One. 2018 Feb 23;13(2):e0193335. doi: 10.1371/journal.pone.0193335 (PMC5825098; doi:10.1371/journal.pone.0193335)
Supplement: S4 Table — (DOCX) [file pone.0193335.s011.docx]

**S4 Table.** GO analysis of genes differentially expressed during N deprivation [ref] and P deprivation (this study).

| **GO process ID** | **Description** | **Similar regulation** | | | | **Unique regulation N** | | | | **Unique regulation P** | | | |
| --- | --- | --- | --- | --- | --- | --- | --- | --- | --- | --- | --- | --- | --- |
|  |  | Down  48 h | Down  72 h | Up  48 h | Up  72 h | Down  48 h | Down  72 h | Up  48 h | Up  72 h | Down  48 h | Down  72 h | Up  48 h | Up  72 h |
| 0009892, 0000105, 0009098, 0033362, 0006564, 0006526, 0008652 | Amino acid biosynthesis | 11 | 18 | 0 | 2 | 0 | 0 | 0 | 0 | 1 | 2 | 0 | 0 |
| 0009083, 0006572 | Amino acid degradation | 0 | 1 | 1 | 3 | 0 | 0 | 0 | 0 | 1 | 1 | 0 | 1 |
| 0006520 | Amino acid metabolism | 4 | 6 | 0 | 3 | 0 | 0 | 0 | 0 | 2 | 2 | 0 | 2 |
| 0006511, 0006511, 0006508, 0016567 | Protein degradation | 5 | 17 | 7 | 18 | 4 | 5 | 3 | 15 | 2 | 7 | 6 | 18 |
| 0036211 | Protein modification | 1 | 5 | 5 | 10 | 0 | 0 | 0 | 0 | 0 | 1 | 1 | 2 |
| 0006422, 0006412, 0006413, 006436, 0006418, 0006457, 0006415, 0006414 | Protein biosynthesis | 103 | 134 | 2 | 2 | 3 | 7 | 0 | 2 | 6 | 15 | 0 | 3 |
| 0006650 | Glycerophospholipid metabolic process | 0 | 0 | 0 | 0 | 0 | 0 | 0 | 0 | 0 | 0 | 2 | 2 |
| 0006629 | Lipid metabolism | 0 | 0 | 1 | 5 | 0 | 0 | 0 | 0 | 0 | 0 | 2 | 4 |
| 0015979, 0016117, 0006779, 0015995, 0009765 | Pigment biosynthesis and photosynthesis | 5 | 44 | 0 | 5 | 3 | 8 | 1 | 1 | 0 | 2 | 0 | 0 |
| 0042255, 0042254, 0006364 | Ribosome biogenesis and assembly | 28 | 28 | 0 | 0 | 0 | 0 | 0 | 0 | 0 | 0 | 0 | 0 |
| 0006094, 0009052, 0006098, 0006006, 0006096, 0005975 | Carbohydrate metabolism | 3 | 16 | 3 | 9 | 1 | 3 | 0 | 3 | 0 | 1 | 4 | 10 |
| 0009113, 0009116, 0006189, 0006164, 0006177, 0006221 | Purine/pyrimidine nucleotide biosynthesis | 10 | 11 | 0 | 0 | 0 | 2 | 0 | 0 | 0 | 1 | 0 | 0 |
| 0009166 | Nucleotide catabolism | 0 | 0 | 2 | 3 | 0 | 0 | 0 | 0 | 0 | 0 | 2 | 2 |
| 0006633 | Fatty acid biosynthesis | 0 | 5 | 0 | 0 | 0 | 0 | 0 | 1 | 1 | 2 | 0 | 0 |
| 0006351, 0006396, 0006352, 0006354 | Transcription | 15 | 20 | 0 | 1 | 1 | 1 | 0 | 1 | 2 | 5 | 0 | 3 |
| 0006355 | Regulation of transcription, DNA-templated | 10 | 14 | 7 | 23 | 1 | 3 | 0 | 13 | 4 | 8 | 4 | 10 |
| 0015986 | ATP synthesis coupled proton transport | 0 | 0 | 0 | 3 | 0 | 0 | 1 | 1 | 2 | 0 | 1 | 3 |
| 0051726 | Regulation of cell cycle | 0 | 1 | 1 | 4 | 0 | 0 | 3 | 2 | 1 | 1 | 1 | 3 |
| 0007165 | Signal transduction | 0 | 1 | 1 | 4 | 0 | 0 | 0 | 1 | 0 | 0 | 0 | 1 |
| 0006807 | Nitrogen compound metabolism | 0 | 0 | 3 | 5 | 0 | 0 | 6 | 6 | 2 | 3 | 1 | 1 |
| 0042128 | Nitrate assimilation | 0 | 0 | 0 | 0 | 0 | 0 | 2 | 2 | 0 | 0 | 0 | 0 |
| 0006777 | Mo-molybdopterin cofactor biosynthesis | 0 | 0 | 0 | 0 | 0 | 0 | 2 | 2 | 0 | 0 | 0 | 1 |
| 0006817 | Phosphate transport | 0 | 0 | 0 | 1 | 0 | 0 | 0 | 0 | 0 | 0 | 6 | 6 |
| 0016311 | Dephosphorylation | 0 | 0 | 1 | 3 | 0 | 0 | 0 | 0 | 0 | 0 | 4 | 4 |
